# Inquiry and Answer

**Published:** 1867-01

**Authors:** 


### INQUIRY AND ANSWER.

Editors of Dental Register:—I heard that the funniest thing that occurred at the Boston meeting was something about a dog. Not having patience to wait for the official report, I have studied that of the "*Dental Cosmos*," to find what constituted the fun, and suppose this is it: (See September "*Cosmos*," page 83).

"Dr. Watt said that periodontal inflammation could nearly always be relieved by a mild course of iodide of potassium. In local effect potassium was pretty nearly nothing as compared with iodine; but chain the two together and they worked powerfully. Iodine by itself will act locally; potassium alone resolves itself into potassa, by its union with oxygen. He compared these agents to two fierce dogs let into the covert among the game, one would seize his prey and hold on to it, while his fellow performed the office of dragging out to the hunter."

I suppose that is the funny thing referred to; if not, I hope the Association will not be "as funny as it can." Fierce dogs those are, and *stout* ones too, when one can hold the game *in* the thicket, *while* the other drags it *out*. Verily that is funny enough, and all the more so when said by Dr. Watt who was born in the woods "with the spotted fawn," where the red deer grazed, and the red man's track was not yet washed out of the soil—said by Dr. Watt whose boyhood was sung to sleep by the baying of hounds, and the brown wolf's howl, and wakened by the shrieks of the screech owl and the noisy courtships of the wild turkey gobbler. Raised thus, and talk so about dogs!

But the chemistry (?) of this is as curious as the dogs. "Chain the two together and they worked powerfully"—that is, iodine and potassium. Now, if my memory is not treacherous, Dr. W. used to tell us in the old college, and the Miss. Valley Association, that iodide of potassium does not work "powerfully," but that locally it is nearly inert, and exerts its influence on the constitution by being decomposed, its elements being thus liberated to act in accordance with their affinities. Am I right?

This may not be the amusing affair referred too, but as it afforded me a good laugh I was satisfied.

QUILL.

### ANSWER.

QUILL'S memory is pretty good; and his criticisms are not without basis. Reporters do not always catch the idea of the speaker; and in our profession chemical remarks are usually left out or misreported without the slightest wrong intention on the part of any one. The following is nearly the "Oral Communication" referred to, and it will be observed that it was on neuralgia, and not on "Periodontal Inflammation."

DR. WATT said, "I do not believe we have time to spend in correcting misapprehensions. If the gentleman insists that there are ignorant dentists, I will come into the ring and show a specimen. Let us go into science. I suppose that neuralgia comes under the head of pathology; I do not know whether the Committee reported on it or not. I presume that the dentists and physicians fail very many times for want of a correct diagnosis of the cause of neuralgia, which is often very obscure. Suppose a ligature were placed around a nerve trunk and suddenly drawn so tight as to cut off all communication. The subject would feel a sudden thrill of pain, and then a numbness—would feel at the time, and afterward, as if the nerve trunk had been severed. But if drawn only so tight as to prevent the free action of the nerves, but not to cut off all communication, there would be intense pain felt at their sentient extremities.

If a surgeon or a dentist has charge of a case of *tic-doloreux*, he will probably take for granted that there is bony pressure on the nerve trunk, and that it is scarcely amenable to treatment. I think that in a great majority of cases there is no such pressure by long deposits, but by thickening of the investing membrane, or of the periosteum that lines the long canal. Such cases are certainly amenable to treatment. Many, in such cases extract teeth, give quinine, calomel, and finally get quit of a troublesome patient by sending him to a watering place. Such cases can nearly always be relieved by a course of iodide of potassium. This is a neutral salt. Its elements have each strong affinities for certain constituents of living tissue, but the salt, as such, has almost no such affinity.

The action of this salt may be illustrated by a common practice of Western hunters. They chain two dogs together till they get to the woods, and then let them loose to start the game. Their powers are not exhausted by previous rambling. So we may chain the iodine and the potassium together and start them into the blood. They do not then cause much local action—are as tame as the chained dogs.

But if we would start the iodine by itself, we would not get much of it into the blood, because it would combine with organic tissues by the way. And potassium by itself would form potassa, by combining with oxygen, and would spend its force in local action. But combine them and they form a salt that can go wherever the blood can go—even where the blood corpuscles can not go. Being neutral and highly soluble, it is readily carried throughout the system; and when there it is gradually decomposed, in obedience to stronger affinities than those holding its elements together. The iodine finds something it likes better than the potassium, and combines with it; and the potassium, without going into mourning, marries oxygen, forming potassa, which is a solvent of all organic tissues. The potassa holding the morbid product in solution, and the iodine combining with them (they being in a more favorable state for chemical action than healthy tissues), they are carried out of the organism by the various emunctories. When the morbid matter is all carried away the disease is cured.

The illustration of the chained dogs may be carried still further. They get fretted and angry at the restraint, and when released, revenge themselves on the game. That is the case with these elements. It is a chemical fact that when any substance is set free from combination it acts with greatly increased energy. So when the iodine reaches the point desired, and is there decomposed, its elements in their nascent condition (like the excited condition of the dogs), act with this increased energy on whatever offers the least resistance. The vitality of morbid tissues being below that of normal ones, offers less resistance to chemical action, and hence they are overcome while the others remain. Of course this is not the real state of the facts, as chemical and vital actions differ; but the dog illustration is given to assist your memories. The potassa uncovers the game, the iodine seizes it (that is, combines with it), and the various emunctories carry it out of the system. I have not tested the perspiration to find whether iodine comes out through the pores of the skin, but I have detected it in the saliva and the secretion of the Schneiderian membrane.

Now my opinion is that when you find a persistent and troublesome case of facial neuralgia, and do not find diseased teeth to explain it, and even if you do, but fail to give relief by treating the teeth, you may suspect pressure on the nerve trunk; and as in but few cases the pressure will result from ossific deposit, relief may be obtained by a course of the iodide, say six to ten grains three times a day.

This is nearly the substance of the remarks referred to, of which "Quill's" quotation is an unintentional caricature. It was made from memory and a small pencil sketch set down as a memorandum while at Boston. Since it was written, I have had, through the kindness of Dr. Shepard, an opportunity to see a copy of the official report of it, and as there is no serious discrepancy, I have concluded to insert it here, for the satisfaction of my friend "Quill," and any others who may wish to see it. To be misreported is a small affair personally considered; but if the speaker has any influence, some might be led into error, which would be a serious matter. Abbreviated reporting is a very difficult task; but when the reporter entirely fails to catch the idea of the speaker, and doesn't understand the subject himself, he should simply hold his pencil till a clear place is reached.

